# Development and Usability Testing of a Web-based COVID-19 Self-triage Platform

**DOI:** 10.5811/westjem.2020.7.48217

**Published:** 2020-08-19

**Authors:** Justin D. Schrager, Keke Schuler, Alexander P. Isakov, David W. Wright, Anna Q. Yaffee, Kara L. Jacobson, Ruth M. Parker, Craig Goolsby

**Affiliations:** * Emory University School of Medicine, Department of Emergency Medicine, Atlanta, Georgia; † National Center for Disaster Medicine and Public Health, Department, Bethesda, Maryland; ‡ Rollins School of Public Health, Department of Health Policy and Management, Atlanta, Georgia; § Uniformed Services University of the Health Sciences, Department of Military and Emergency Medicine, Bethesda, Maryland

## Abstract

**Introduction:**

The development and deployment of a web-based, self-triage tool for severe respiratory syndrome coronavirus 2 (COVID-19 disease) aimed at preventing surges in healthcare utilization could provide easily understandable health guidance with the goal of mitigating unnecessary emergency department (ED) and healthcare visits. We describe the iterative development and usability testing of such a tool. We hypothesized that adult users could understand and recall the recommendations provided by a COVID-19 web-based, self-triage tool.

**Methods:**

We convened a multidisciplinary panel of medical experts at two academic medical schools in an iterative redesign process of a previously validated web-based, epidemic screening tool for the current COVID-19 pandemic. We then conducted a cross-sectional usability study over a 24-hour period among faculty, staff, and students at the two participating universities. Participants were randomly assigned a pre-written health script to enter into the self-triage website for testing. The primary outcome was immediate recall of website recommendations. Secondary outcomes included usability measures. We stratified outcomes by demographic characteristics.

**Results:**

A final sample of 877 participants (mean age, 32 years [range, 19–84 years]; 65.3% female) was used in the analysis. We found that 79.4% of the participants accurately recalled the recommendations provided by the website. Almost all participants (96.9%) found the website easy to use and navigate.

**Conclusion:**

Adult users of a COVID-19 self-triage website, recruited from an academic setting, were able to successfully recall self-care instructions from the website and found it user-friendly. This website appears to be a feasible way to provide evidence-based health guidance to adult patients during a pandemic. Website guidance could be used to reduce unnecessary ED and healthcare visits.

## INTRODUCTION

The severe respiratory syndrome coronavirus 2 (COVID-19 disease) pandemic has led to more than 287,399 deaths worldwide.^1^ Hospitals in the United States are experiencing surges of patients and critical shortages of personal protective equipment (PPE).^2^ Because most hospitals routinely operate near capacity, these patient surges could lead to inadequate patient care and an insufficient number of beds, treatment spaces, and healthcare workers to evaluate patients.^3^ Each unnecessary patient visit creates additional potential COVID-19 exposures to healthcare workers and other vulnerable patients in the hospital, and consumes scarce PPE.

A key strategy to mitigate the healthcare system burden is “forward triage.”^4^ For this strategy to work, a patient must be empowered to make informed decisions about the need for emergency care services, with the ultimate goal of safely reducing unnecessary emergency department (ED) visits.^4^ A web-based, free, educational platform, can fulfill this strategy by providing immediate instructions for next steps in care and information on potential testing sites, thereby preventing unnecessary healthcare worker exposure and exposure of other vulnerable patients to COVID-19, while conserving PPE.^4^,^5^

Web-based self-triage was first developed and deployed during the 2009 novel influenza A (H1N1) virus pandemic by researchers at Emory University. The evidence-based Strategy for Offsite Rapid Triage (SORT) was rapidly validated and integrated into web platforms hosted on the US Department of Health & Human Services flu.gov website as well as Microsoft Corporation’s H1N1 Response Center.^6^ The sites recorded more than 670,000 completed self-evaluations over five months during the outbreak. Success with the H1N1 self-triage website provided the groundwork for a public COVID-19 self-triage website (www.c19check.com). The original SORT algorithm has been updated to reflect the best available evidence about COVID-19 as shared by the Centers for Disease Control and Prevention and the World Health Organization, and was built iteratively with multidisciplinary input from experts in infectious disease, emergency medicine, pre-hospital medicine, epidemiology, and health literacy. On the website the user enters age, ZIP code, comorbidities, and symptoms, and the algorithm classifies risk as low, intermediate, or high (Figure). The website then provides the user CDC-based recommendations and level of risk as well as ZIP Code-specific local health department information if the user chooses to enter that piece of personal information. Widespread adoption of an effective self-triage tool has the potential to mitigate unnecessary ED visits.

### Objective

The primary objective of this study was to determine whehter participants understood and recalled recommendations provided by a COVID-19 web-based, self-triage tool.

## METHOD

### Design

Investigators at Emory University and Uniformed Services University of the Health Sciences performed usability testing of the COVID-19 self-triage website. The institutional review boards approved this study and waived the consent requirement; however, an assent script with affirmation was used. On March 19–20, 2020, staff, faculty, and students at both institutions were recruited via email for voluntary study participation. Participants received an email with instructions and a link to the self-triage website. After completing demographic information, each participant was randomly assigned a pre-written patient “script” that the participant referred to when using the COVID-19 checker website. Once participants inputted scripted information into the COVID-19 checker website and received a recommendation, they were directed to complete the user-feedback form. This form contained questions about their understanding of the recommendations, and their subjective experiences using the website.

We excluded participants’ responses if they did not complete all fields in the website or the user- feedback form. The website is currently designed to triage adult patients. To test the software, some participants were randomly assigned clinical scripts with patients younger than 18 years old. These cases were not allowed to progress to the clinical questions or website guidance and were therefore removed prior to endpoint analysis.

### Setting

The study occurred virtually with participants at Emory University and Uniformed Services University of the Health Sciences.

### Participants

Staff, students, and faculty at Emory University and Uniformed Services University of the Health Sciences participated in the study.

### Main Outcome Measures

We assessed participants’ ability to immediately recall the specific self-care instructions provided by the site, scored as either correct or incorrect. Self-care instructions were specific and nuanced, depending on the symptoms and comorbidities of the scripted case provided. Secondary outcomes were participant feedback about website usability.

## RESULTS

A total of 926 participants enrolled in the study. One participant was removed for being younger than 18 years old, and participants who received pediatric scripts (n = 46) or missing scripts information (n = 2) were removed from analysis, leaving 877 participants (mean age, 32 years [range, 19–84 years]; 65.3% female) in the final analyses (Table). We found that 79.4% of the participants accurately recalled the instructions and guidance provided by the website, and 96.9% of the participants reported that the website was “easy” or “very easy” to use (responses of 4-“easy” or 5-“very easy” on a Likert scale). Responses were further broken down based on demographic characteristics.

## DISCUSSION

This study demonstrates the feasibility of a web-based, self-triage tool to provide information to adults seeking guidance about COVID-19 symptoms and next steps. Approximately four out of five participants correctly recalled and identified the website’s recommendations, and nearly all participants described the website as easy to use.

The sampling methodology allowed for rapid feedback of the website, and free-text suggestions were expeditiously incorporated into the website. Suggested modifications were incorporated the day after study enrollment, and the website was launched for public use the following day. Using the prior H1N1 website experience, the current COVID-19 algorithm was created, rapidly iterated, adapted, tested, and electronically deployed within days of sustained community transmission in the US, and well before the anticipated zenith of COVID-19 in the US.^1^ This suggests that it is also feasible to maintain and quickly update this platform for future pandemic response. This project was conducted collaboratively between Emory University and the private company Vital Software, Inc., an innovation development partner of Emory University that provided the technical expertise and hosting at no cost.

Thus far, there is high public demand for this self-triage tool. Between March 26–May 14, 2020, C19check.com has hosted 766,574 unique visitors and completed 395,895 self-assessments. In comparison, there were 320,333 unique visits to the H1N1 self-triage website during its first three months. The self-triage algorithm will be updated in real time as new guidance and data are published by national and international public health experts, and in continued consultation with health literacy experts. Future studies will validate the algorithms and test user understanding of self-assessment instructions, adherence to instructions, and intent-to-use healthcare resources before and after self-triage within the wider population of website users. Website data will also be used to map epidemiologic patterns of disease and symptoms and will continue to expand upon the list of 15 foreign-language translations available.

## LIMITATIONS

The participants were students and faculty at two academic institutions and thus the results cannot be applied to the general population due to mismatched health literacy and recall capacity. Given the exigencies of the COVID-19 pandemic, these sites were chosen since a large number of participants could be recruited rapidly, and the free-text feedback from medically-oriented participants was instructive for the design team. For this reason, we chose to capture a mix of free-text usability feedback and Likert-scale usability scores rather than a validated usability scale for the secondary outcome. This study tested immediate recall of instructions, rather than lasting recall of instructions, as the website was designed to be used by a person actively making the decision to seek medical care or not, and the instructions would be less relevant at a later date.

Additionally, the self-care instructions were nuanced and specific by design, which could have negatively impacted recall success depending on the comorbidities and symptoms in the user’s assigned scripted case. Further, the scripted nature of the cases could also have had a negative impact on recall success. This study did not seek to identify adherence to the instructions (eg, behavior change) since the participants were not actually using their own real patient data. The study did not assess the outcomes of actual COVID-19 patients following recommendations from the site.

## CONCLUSION

This study demonstrates that adult users in an academic setting can correctly identify recommended care instructions from a self-triage website during a pandemic. Study participants found the website user-friendly. The website was adapted from a pre-existing, self-triage algorithm in an iterative, expeditious manner. To date, there has been high demand for the website, and it has potential to provide users valuable health information and mitigate unnecessary ED visits. Limiting unnecessary healthcare visits will benefit both patients and healthcare workers by reducing COVID-19 exposure while conserving scarce resources.

## Supplementary Information



## Figures and Tables

**Figure:**
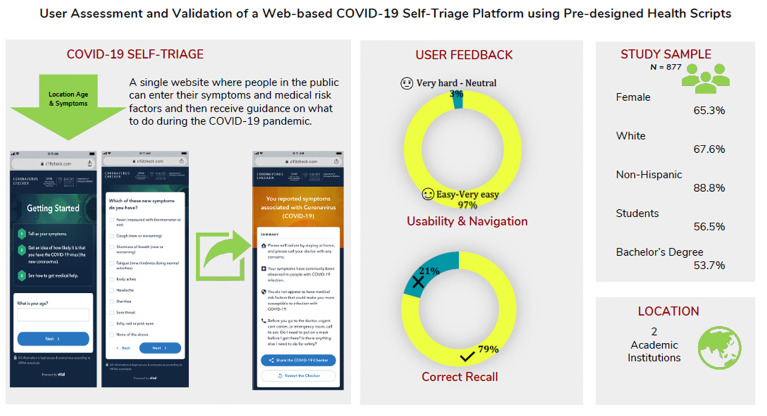
Visual abstract of user interface and experience as well as usability testing.

**Table:** Sample demographic characteristics of 877 participants who completed COVID-19 checker testing and user-feedback responses by sample characteristics.

Sample demographic characteristics*	No. (%) of participants	No. (%) of participants recalled correctly within characteristic
Gender		
Female	573 (65.3)	467 (81.5)
Male	273 (31.1)	213 (78.0)
Race		
White	593 (67.6)	487 (82.1)
Asian	138 (15.7)	97 (70.3)
Black or African American	64 (7.3)	51 (79.7)
Native American or Alaska Native	2 (0.2)	0 (0)
Hawaiian or other Pacific Islander	1 (0.1)	1 (100)
Ethnicity		
Hispanic	67 (7.6)	59 (88.1)
Non-Hispanic	779 (88.8)	620 (79.6)
Employment		
Student, full-time	496 (56.5)	407(82.2)
Employed, full-time	304 (34.7)	238 (78.3)
Student, part-time	7 (0.8)	5 (71.4)
Employed, part-time	20 (2.3)	15 (75.0)
Homemaker	5 (0.6)	4 (80.0)
Caregiver	2 (0.2)	1 (50.0)
Full-time volunteer	2 (0.2)	2 (100)
Annual Income		
$0	154 (17.6)	124 (70.5)
$1 to $9,999	115(13.1)	92 (80.0)
$10,000 to $24,999	86 (9.8)	67 (77.9)
$25,000 to $49,999	135 (15.4)	115 (85.2)
$50,000 to $74,999	68 (7.8)	52 (76.5)
$75,000 to $99,999	65 (7.4)	52 (80.0)
$100,000 to $149,999	61 (7.0)	47 (77.0)
$150,000 and greater	60 (6.8)	49 (81.7)
Highest Education		
Bachelor degree	471 (53.7)	383 (81.3)
Master degree	164 (18.7)	128 (78.0)
Doctorate degree	155(17.7)	128 (82.6)
Professional degree	26 (3.0)	20 (76.9)
Some college credit, no degree	11 (1.3)	9 (81.8)
High school graduate	7 (0.8)	5 (71.4)
Associate degree	4 (0.5)	1 (25.0)
Some high school, no diploma	1 (0.1)	0 (0)

* Categories: Unknown, prefer not to answer, and other are not listed.

## References

[1] World Health Organization (2020). Coronavirus disease 2019 (COVID-19) Situation Report – 114.

[2] Giwa AL, Desai A, Duca A (2020). Novel 2019 coronavirus SARS-CoV-2 (COVID-19): an updated overview for emergency clinicians. Emerg Med Pract.

[3] Willan J, King AJ, Jeffery K (2020). Challenges for NHS hospitals during covid-19 epidemic. BMJ.

[4] Hollander JE, Carr BG (2020). Virtually perfect? Telemedicine for Covid-19. N Engl J Med.

[5] Greenhalgh T, Wherton J, Shaw S (2020). Video consultations for Covid-19. BMJ.

[6] Kellermann AL, Isakov AP, Parker R (2010). Web-based self-triage of influenza-like illness during the 2009 H1N1 influenza pandemic. Ann Emerg Med.

