# Supplementary material for: Development and Usability Testing of a Web-based COVID-19 Self-triage Platform
Source: West J Emerg Med. 2020 Aug 19;21(5):1054–8. doi: 10.5811/westjem.2020.7.48217 (PMC7514387; doi:10.5811/westjem.2020.7.48217)
Supplement: Supplementary file 1 [file wjem-21-1054-s001.docx]

Survey Instrument

(Mar 19, 2020)

1. What is your age?
2. What is your gender?

-Male

-Female

-Non-binary

-Prefer not to say

-Other (write in)

1. What is your race?

-White

-Black or African American

- American Indian/Alaskan Native

-Asian

-Native Hawaiian/Pacific Islander

-Other

1. What is your ethnicity?

-Hispanic

-Non-Hispanic

1. Which of these options best describes your current employment status?

-Employed Part Time (less than 40 hours)

-Employed Full-Time (40 hours or greater)

-Full time volunteer

-Caregiver (eg. children or elderly)

-Homemaker

-Student, full-time

-Student, part-time

-Other

-Prefer not to say

1. Which of these options best describes your household income this past year?

-$0

-$1-$9,999

-$10,000 to $24,000

-$25,000 to $49,999

-$50,000 to $74,999

-$75,000 to $99,999

-$100,000 to $149,999

-$70,001-$90,000

-$150,000 or greater

1. What is the highest degree or level of school you have completed?

-No schooling completed

-Nursery school to 8^th^ grade

-Some high school, no diploma

-High school graduate, diploma, or the equivalent (GED)

-Some College/Some Technical School no degree

-Associate Degree

-Bachelor’s Degree

-Master’s Degree

-Professional Degree

-Doctorate Degree

-Prefer not to say

**Please complete the Symptom Tracker and then answer the following questions**

1. Did you understand the instructions at the end of the website when it gave you information about your risk for COVID-19 and information on next steps to take?

- Yes

- No

- Maybe

1. Please select the instructions that the website gave you at the end?

- It is unlikely you have Coronavirus (COVID‑19): Avoid close contact with people who are sick and practice social distancing.
- It is possible you have Coronavirus (COVID‑19): Please self isolate by staying at home.
- It is possible you have Coronavirus (COVID‑19): Please self isolate by staying at home. Because you have other medical conditions, we recommend you call your doctor.
- We recommend that you seek medical attention by calling your doctor. If you are having an emergency, please call 9-1-1 or go to the ER.
- Not Sure or Don't remember

9. Did you find anything confusing on the website?

- [Open ended answer]

10. Are there any specific grammar or wording recommendations you believe we could improve?

- [Open ended answer]

11. How easy was the website to use and navigate?

- (very easy) 5– 4 – 3 -2- 1 (very hard)
